# Effect of novel graphitic carbon/NiO hole transporting electrode on the photovoltaic and optical performance of semi-transparent perovskite solar cells†

**DOI:** 10.1039/d2ra08198a

**Published:** 2023-03-06

**Authors:** Shubhranshu Bhandari, Sreeram Valsalakumar, Yusuf Chanchangi, Prabhakaran Selvaraj, Tapas K. Mallick

**Affiliations:** a Environment and Sustainability Institute, University of Exeter Cornwall UK TR10 9FE s.bhandari@exeter.ac.uk shubhranshu0094@gmail.com; b Wolfson School of Mechanical, Electrical and Manufacturing Engineering, Loughborough University UK LE11 3TU

## Abstract

Perovskite devices can play a critical role as tunable semi-transparent photovoltaics managing the buildings' energy health for energy harvesting, storage and utilization. Here we report ambient semi-transparent PSCs with novel graphitic carbon/NiO-based hole transporting electrodes having variable thicknesses achieving a highest efficiency of ∼14%. On the other hand, the altered thickness produced the highest average visible transparency (AVT) of the devices, nearly 35%, which also influenced other glazing-related parameters. This study envisages the impact of the electrode deposition technique on indispensable parameters like colour rendering index, correlated colour temperature, and solar factor evaluated using theoretical models to illuminate these CPSCs' colour and thermal comfort for BIPV integration. The solar factor value between 0 to 1, CRI value >80 and CCT value >4000 K make it a significant semi-transparent device. This research work suggests a possible approach to fabricating carbon-based PSC for high-performance semi-transparent solar cells.

## Abstract 2

Perovskite devices can play a critical role as tunable semi-transparent photovoltaics managing the buildings' energy health for energy harvesting, storage and utilization.

## Graphical abstract

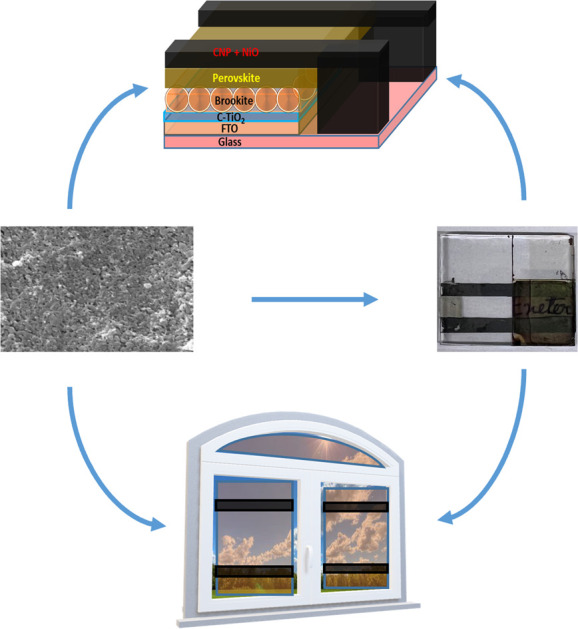

## Introduction

Perovskite solar cells (PSCs) have explored great breakthroughs in the field of third-generation solar cells and spurred researchers to develop a variety of new architectures.^1–19^ These cells mainly consist of electron transport-spacer/perovskite-hole transport layers, a simple fabrication process exhibiting high power conversion efficiency (PCE) and enhanced stability towards environmental factors.^20–24^ Although highly efficient, PSCs sometimes rapidly lose their efficiency due to the hygroscopic and air-sensitive character of the materials used.^25^ Therefore, the selection of materials and their fabrication process has a significant role in the performance. PSCs often include a carbon-based back contact, a suitable solution to substitute noble metals due to their high conductivity, low cost, low-temperature processing, work function close to gold, and ambient stability.^26–33^ So far, various carbon polymorphs and composites have been used for PSCs as counter electrodes.^34–40^ The property of the counter electrode is highly crucial to determine the semi-transparent nature of PSCs as well.^41–46^ In this context, maintaining healthy optical and photovoltaic characters using carbon-based electrodes can be a game-changer that has not been thoroughly explored to date.

Here we report the utilization of novel graphitic carbon nanoparticles (CNP) synthesized from plant materials and NiO as a hole transporting electrode (HTE) having variable thicknesses for CH_3_NH_3_PbI_3_ based PSCs with an FTO/compact TiO_2_/brookite TiO_2_/perovskite/graphitic CNP–NiO architecture. The method is based on a fully wet deposition process, which takes less time and utilizes a combination of spin-coating and blade-coating methods. The thickness of the HTE was varied by 1-step to 3-step deposition with a sheet resistance variation of ∼12 to ∼25 ohm. In connection, an impressive photovoltaic parameter with an overall average PCE of ∼11% was achieved for these carbon-based devices. The influence of thickness significantly altered the AVT (average visible transmittance) of the devices as well attaining an overall average of ∼31%, which influenced the calculation of the glazing-related parameters like correlated colour temperature (CCT), colour rendering index (CRI), solar factor (SF), and subjective rating (SR). We believe this finding reports an excellent combination of photovoltaics and optical performances of graphitic carbon-based PSC (CPSC) *via* thickness engineering as a futuristic approach for building-integrated photovoltaics.

## Materials and methods

The synthesis of graphitic CNP (carbon nanoparticles) and device fabrication methods were adopted from our earlier reported article.^47^ The entire device fabrication process was carried out under ambient conditions for all cases. In short, fluorine doped tin oxide (FTO) glass substrate (2 cm × 2 cm) was etched, followed by standard cleaning procedures in the first place as described in ESI.† Next, 0.35 ml titanium isopropoxide (TTIP) in 0.1 ml of 2 M HCl and 5 ml ethanol was used as the precursor solution of the blocking-TiO_2_ layer. The compact-TiO_2_ layer was spin-coated at 3000 rpm for 30 s, then heated at 415 ± 10 °C for 30 minutes and cooled to room temperature. After that, the brookite TiO_2_ (3 wt% aqueous suspensions) layer was coated following our previous report, followed by heating at 150 °C for 30 min.^48^ Next, the MAPI (MAI and PbI_2_ were mixed in a 1 : 1 molar ratio) precursor solution (4 : 1 DMF : DMSO) with an appropriate amount (50 μl) was spin-coated at 1000 and 5000 rpm for 10 s and 20 s, respectively. During the last 10 s of rotation, chlorobenzene (400 μl) was splashed from the top. Then the devices were heated at 100 °C for 10 min and cooled down to room temperature. Finally, the low-temperature carbon/NiO composite HTE was deposited by screen printing and heated at 100 °C for 10 min (this step was done 1 to 3 times). The carbon paste was prepared by mixing graphite (Aldrich; product number: 282863) and graphitic carbon nanoparticles (2 : 1 w/w) uniformly in ∼8 g terpineol *via* ball milling for 2 h. Then, 1.8 ml TTIP, 0.2 ml Hac (glacial acetic acid), 5 ml ethanol and 2.5 g NiO (Sigma-Aldrich; Product code: 637130) were added to the mixture by ball milling for another 10 h to gain homogenized carbon paste.

The theoretical calculations for the optical performance of devices have been detailed in the ESI.†

## Results and discussions

**Fig. 1:**
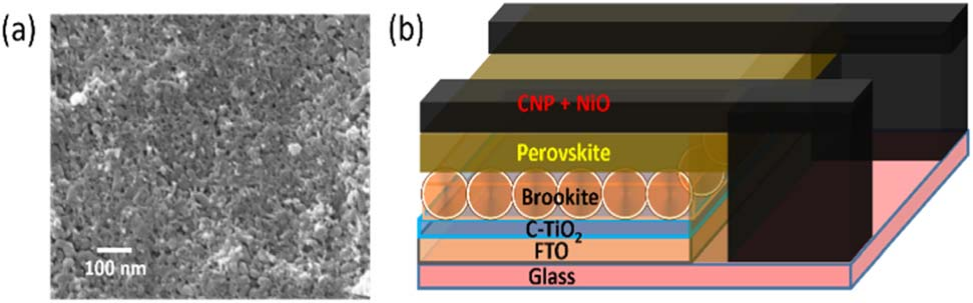
(a) Top surface SEM of carbon electrode and (b) schematic diagram of the semi-transparent PSC.

The top surface SEM (scanning electron microscopy) of the CNP–NiO hole transporting system coated on top of a glass substrate is shown in Fig. 1a. The particle nature is very clear from the SEM, showing particle size in the range of 40 to 70 nm for the CNP and NiO. The hole transport property of NiO was the reason behind its inclusion in the CNP paste. The Device structure is shown in Fig. 1b, which indicates the small carbon bar technique to utilize the CPSC as the semi-transparent device. Depending on the number of carbon layer printing steps, the performances of the devices were analyzed. Each printing step produces ∼2 microns thick carbon layer. The sheet resistance measurement was carried out for the carbon layer depending on the number of printing steps using the Ossila four-point probe system. Table S1, ESI† shows the nature of sheet resistance according to the coating steps. Lower sheet resistance was desirable as it implies higher conductivity for the better performance of perovskite devices. These measurements helped in the understanding of the cell fabrication process. These devices consist of brookite TiO_2_ as an effective electron transport layer instead of high-temperature mesoporous layers, which also reduces the time and cost of fabrication. Also, brookite TiO_2_ and perovskite make better interfacial contact, according to previous literature.^48^

**Fig. 2:**
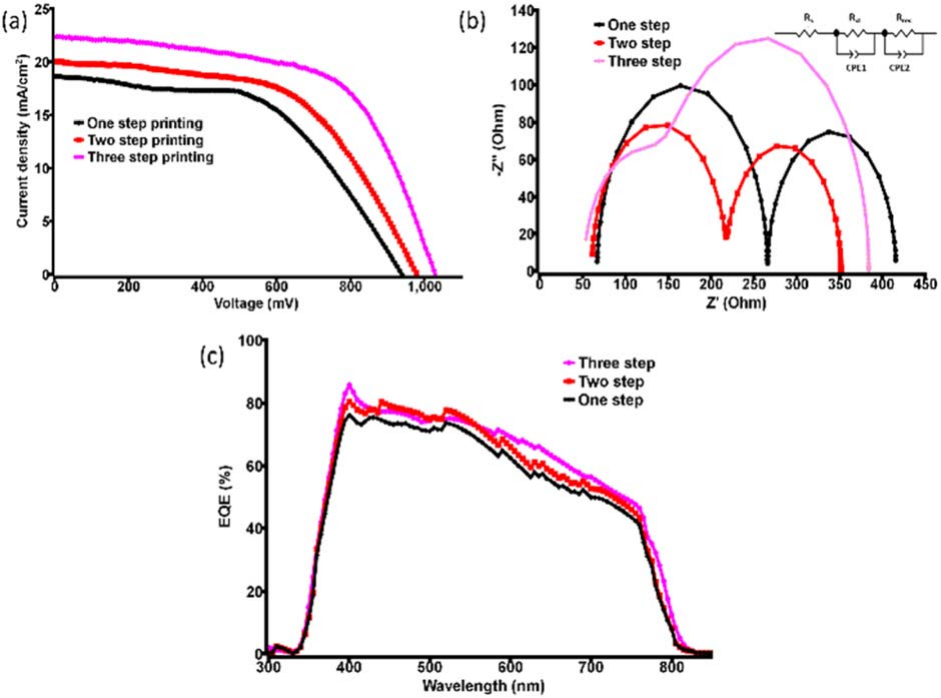
(a) Current density *vs.* voltage (*J*–*V*) plot for champion devices with different printing steps in reverse bias, (b) corresponding EIS spectra of the corresponding champion devices (inset: circuit diagram) and (c) IPCE measurement of the devices indicating high external quantum efficiency.

The fabricated devices were initially tested with cross-sectional SEM to understand the layer thickness. The brookite and perovskite layers had thicknesses of ∼200 and ∼400 nm, respectively (Fig. S1, ESI†). Next, the photovoltaic performance was determined using the solar simulator and *I*–*V* tracer. The current density *vs.* voltage (*J*–*V*) characteristic measurement was performed under simulated AM 1.5 (100 mW cm^−2^) for the three different types of devices, as shown in Fig. 2a. Table 1 provides information about efficiency, short circuit current density (*J*_SC_), open-circuit voltage (*V*_OC_) and fill factor (FF) of the champion CPSCs of each set considering an active area of 0.12 cm^2^ by masking in reverse bias condition. Six devices were fabricated for each set in a batch to visualize the reproducibility of the results, as shown in Fig. S2b, ESI.† The hysteresis effect on the photovoltaic performance for champion devices has been displayed in Fig. S3, ESI,† which suggests minimal changes in forward and reverse bias conditions making the cells efficient. The average photovoltaic performances of devices are given in Table S2, ESI.† The one-step printed devices showed average PCE values of 8.5%. The two-step printed devices showed an average PCE of 10.3%. In the case of three-step coated CPSCs, an average PCE of 12.7% was observed. The effect of NiO and CNP for the devices were also observed by developing PSCs without NiO and CNP, as illustrated in Fig. S4, ESI.† The results imply maximum PCE of 10.5% and 12% for devices without NiO and CNP, respectively. Similarly, the effect of 4 step coating shows similar average PCE for the devices which implies three step coating as the saturation point.

**Table 1:** Photovoltaic parameters (reverse bias) of champion CPSCs under 1 SUN 1.5 AM, with an active area of 0.12 cm^2^ using photomask

Printing step	*J*_SC_ (mA cm^−2^)	*V*_OC_ (mV)	FF (%)	PCE (%)	Integrated *J*_SC_ from IPCE (mA cm^−2^)
One step	18.65	935.4	55.5	9.68	17.0
Two step	20.01	980.6	58.2	11.4	18.5
Three step	22.39	1028.5	60.8	14.0	20.6

Further, the electrochemical impedance spectroscopy (EIS) measurements were carried out to understand the charge transport properties at different interfaces. The EIS spectrum (Nyquist plot) with the equivalent circuit diagram of the concerned CPSCs was recorded under dark at 0.9 V bias from 1 MHz to 10 mHz, as shown in Fig. 2b. The EIS analysis showed two semi-circle systems in the Nyquist plot. By using EC-lab software, the best fit was obtained for the given circuit diagram (inset Fig. 2b).

In the circuit diagram, *R*_S_ represents the series resistance, including the resistance of FTO and HTE systems. *R*_CT_ is the charge transfer resistance at the perovskite/carbon interface, and *R*_rec_ is the charge recombination resistance at TiO_2_/MAPbI_3_ interface.^17^ The small parabola in the high-frequency region for the three-step printed device indicates lower charge exchange resistance from perovskite to carbon counter electrode (HTE), enhancing the fill factor as reflected from *J*–*V* characterization. On the other hand, the large *R*_rec_ value implies a slow charge recombination process or a low charge recombination rate. This low recombination rate is responsible for high values of *J*_SC_ and *V*_OC_, which are reflected in the *J–V* curves. Devices with higher *R*_S_ values should have lower efficiency, which can be observed in Table 2.

**Table 2:** EIS spectra fitting data of champion CPSCs

Printing step	*R*_S_ (ohm)	*R*_CT_ (ohm)	*R*_rec_ (ohm)
One step	67.1	198.8	149.8
Two step	60.74	133.9	156.7
Three step	51.8	94.14	238.0

**Fig. 3:**
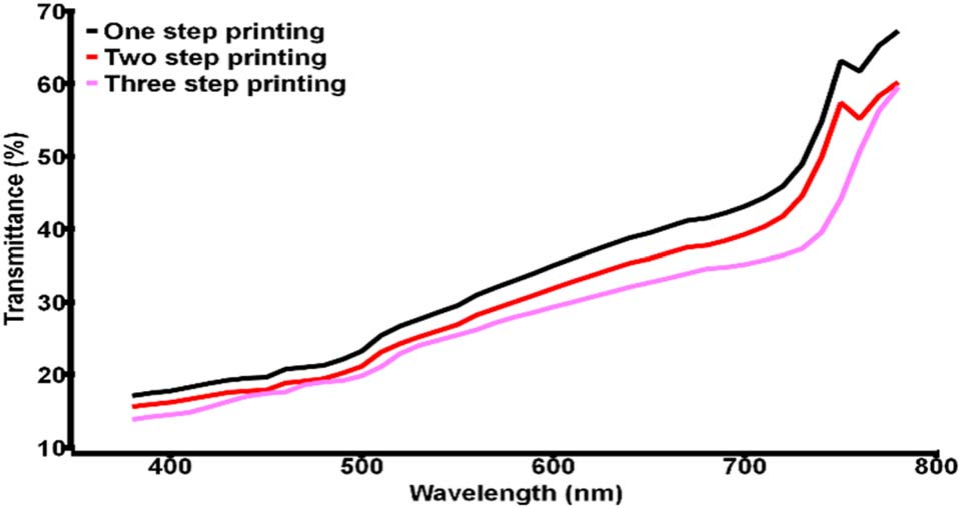
Transmittance *vs.* wavelength plot for the champion devices with various printing steps.

**Fig. 4:**
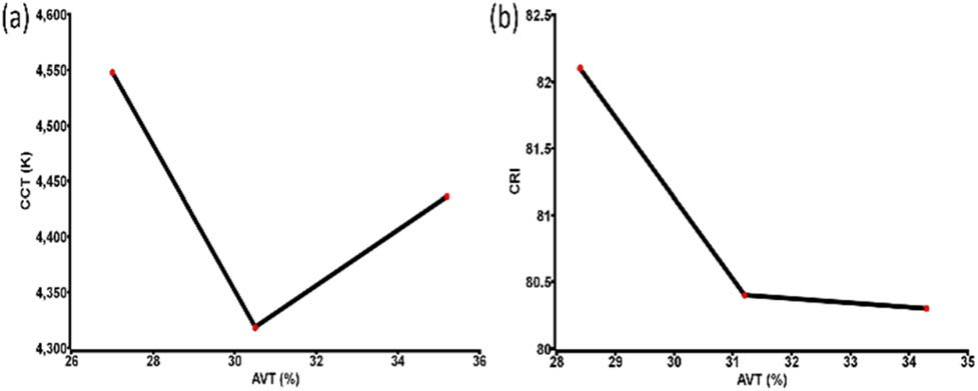
(a) CCT *vs.* AVT and (b) CRI *vs.* AVT of the champion devices fabricated by variable electrode deposition steps.

The other important factor in confirming the nature of devices was incident photon to electron conversion efficiency (IPCE) determination. It showed high external quantum efficiency (EQE) for the devices, as displayed in Fig. 2c and the integrated current density values closely matched with *J*–*V* data. Fig. 2c also implies nice IPCE coverage in the range of 400 to 700 nm for different champion devices indicating good perovskite film quality. To clarify the reliability of the cell performance box and whiskers plot of the PCE for each type of device is given in Fig. S2, ESI.† After realizing the device's photovoltaic properties, the semi-transparent nature of the CPSCs was examined. The transmittance of the different sets of devices is shown in Fig. 3 (data obtained from UV-visible spectrophotometer), which implies the highest AVT for the single-step printed devices. The AVT values observed for the champion device of each set are given in Table 3. An impressive value of 34.3% was noticed for the single-step coating of the counter electrode in CPSC, although the photovoltaic performances of these devices are the poorest. AVT of devices without any carbon bar HTE system was also observed to understand the difference, which showed an impressive value of >50%. The inclusion of three layers severely damages the AVT of the devices, although they have produced better photovoltaic data. Next, different glazing-related parameters were calculated for these CSPCs using theoretical models and equations following previous literature.^42^ CCT and CRI are two important parameters for glazing purposes. Typically a CCT value between 3000 and 7000 K is suitable for entering daylight through glazing. On the other hand, a CRI value near 100 is highly favourable, although values ≥80 are also acceptable. The observed CCT and CRI values for prepared CPSCs are fascinating, as shown in Fig. 4. The highest CCT and CRI were observed for the devices with lower AVT, *i.e.* the triple-printed devices showed higher CCT and CRI along with high PCE values as well (Table 3).

**Table 3:** AVT, CCT, CRI and SF values of the champion devices

Printing step	AVT (%)	CCT (K)	CRI	SF
One step	34.3	4436.09	80.3	0.57
Two step	31.2	4318.52	80.4	0.53
Three step	28.4	4547.68	82.1	0.52

**Fig. 5:**
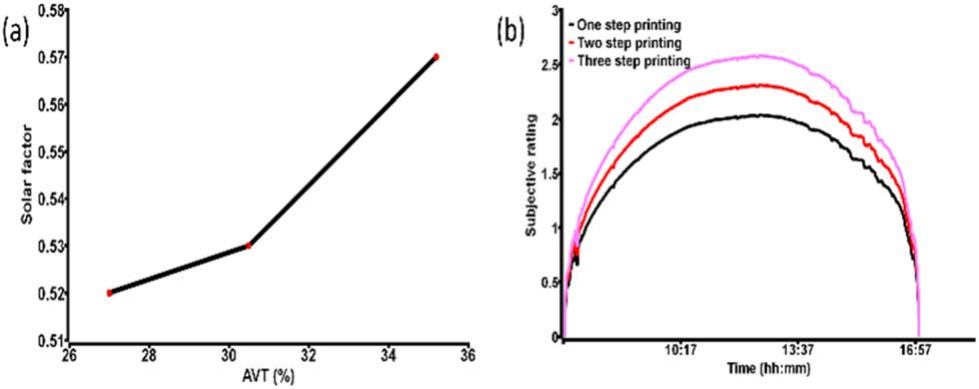
(a) SF *vs.* AVT of the champion CPSCs considering each type and (b) SR of the champion devices considering a clear sunny day of Cornwall in Summer.

Further, the SF and SR of the devices were evaluated. The SF value indicates the protection parameter from the solar radiation, which is usually between 0 to 1. The value near zero implies the best protection from solar radiation. The SF values significantly suggest the lowest value for the lower AVT based devices pointing to more suitable protection from solar radiation. Table 3 and Fig. 5a give the idea of the SF parameter. The trends reflect convenient colour comfort as well as radiation control for the devices having lower values of AVT and higher PCE.

Finally, the SR of the CPSCs were considered taking into account the clear sunny day of Cornwall during the summer, as shown in Fig. 5b. SR dictates the glare daylight control potential, and values ≥2.5 become intolerable. The fabricated devices showed the potential for daylight control during the clear sunny day of summer, which makes them highly suitable for cloudy or intermittent cloudy conditions.

Noticeably the SR value is near the acceptable range for the devices with higher AVT. Although high PCE devices have better protection from solar radiation, they lack daylight control through glazing. This indicates the difficulty of attaining colour comfort and glare daylight control potential in the same device.

Finally, the stability of the devices was observed under ambient conditions kept in the dark. Devices with one-step coating were found to be less stable compared to three-step CNP coating. Usually, carbon as a counter electrode protects devices from atmospheric (air and moisture mainly) interaction; thus, the thickness of the HTE produced the differences in stability, as shown in Fig. S5, ESI.† The maximum stability of three-step carbon-coated devices was ∼600 h with a loss of approximately 10% of initial efficiency.

## Conclusion

In this work, semi-transparent CPSCs were fabricated under ambient conditions, and performances (photovoltaic and optical) were investigated depending on the electrode printing technique. Low-temperature graphitic CNP–NiO HTE system significantly influenced the performances. The AVT value of 34.3% for CPSC makes this study significant, although the device with the highest PCE (14%) attained the lowest value of AVT (28.4%). Optical parameters like CCT, CRI, SF and SR have shown remarkable values that dictate the carbon-based device's potential. This study is noteworthy for more detailed future research to unveil the possibilities of CPSC for building integrated photovoltaic systems by incorporating low-temperature procedures to save time and cost.

## Author contributions

Shubhranshu Bhandari: Conceptualization, Methodology, Formal analysis, Investigation, Writing – original draft, Visualization. Sreeram Valsalakumar: Investigation, Writing – review & editing, Visualization. Yusuf Chanchangi: Investigation, Writing – review & editing, Visualization. Prabhakaran Selvaraj: Investigation, Writing – review & editing, Visualization. Tapas Mallick: Writing – review & editing, Visualization, Supervision, Project administration, Funding acquisition.

## Conflicts of interest

There are no conflicts to declare.

## Supplementary Material

RA-013-D2RA08198A-s001

## References

[cit1] Huang G., Wang C., Zhang H., Xu S., Xu Q., Cui Y. (2018). J. Mater. Chem. A.

[cit2] Rong Y., Hou X., Hu Y., Mei A., Liu L., Wang P., Han H. (2017). Nat. Commun..

[cit3] Kim D. H., Han G. S., Seong W. M., Lee J.-W., Kim B. J., Park N.-G., Hong K. S., Lee S., Jung H. S. (2015). ChemSusChem.

[cit4] Seo J.-Y., Uchida R., Kim H.-S., Saygili Y., Luo J., Moore C., Kerrod J., Wagstaff A., Eklund M., McIntyre R., Pellet N., Zakeeruddin S. M., Hagfeldt A., Grätzel M. (2018). Adv. Funct. Mater..

[cit5] Zhu L., Shao Z., Ye J., Zhang X., Pan X., Dai S. (2016). Chem. Commun..

[cit6] Kim B., Ko S. G., Sonu K. S., Ri J. H., Kim U. C., Il Ryu G. (2018). J. Electron. Mater..

[cit7] Chen W., Yin X., Que M., Xie H., Liu J., Yang C., Guo Y., Wu Y., Que W. (2019). J. Power Sources.

[cit8] Kim H.-S., Lee C.-R., Im J.-H., Lee K.-B., Moehl T., Marchioro A., Moon S.-J., Humphry-Baker R., Yum J.-H., Moser J. E., Grätzel M., Park N.-G. (2012). Sci. Rep..

[cit9] Wang Z., Fang J., Mi Y., Zhu X., Ren H., Liu X., Yan Y. (2018). Appl. Surf. Sci..

[cit10] Cao K., Zuo Z., Cui J., Shen Y., Moehl T., Zakeeruddin S. M., Grätzel M., Wang M. (2015). Nano Energy.

[cit11] Liu S., Huang W., Liao P., Pootrakulchote N., Li H., Lu J., Li J., Huang F., Shai X., Zhao X., Shen Y., Cheng Y.-B., Wang M. (2017). J. Mater. Chem. A.

[cit12] Yang Y., Ri K., Mei A., Liu L., Hu M., Liu T., Li X., Han H. (2015). J. Mater. Chem. A.

[cit13] Mali S. S., Kim H., V Patil J., Hong C. K. (2018). ACS Appl. Mater. Interfaces.

[cit14] Park N.-G. (2013). J. Phys. Chem. Lett..

[cit15] Ku Z., Rong Y., Xu M., Liu T., Han H. (2013). Sci. Rep..

[cit16] Liu T., Xiong Y., Mei A., Hu Y., Rong Y., Xu M., Wang Z., Lou L., Du D., Zheng S., Long X., Xiao S., Yang S., Han H. (2019). RSC Adv..

[cit17] Liu S., Cao K., Li H., Song J., Han J., Shen Y., Wang M. (2017). Sol. Energy.

[cit18] Lee M. M., Teuscher J., Miyasaka T., Murakami T. N., Snaith H. J. (2012). Science.

[cit19] Giordano F., Abate A., Correa Baena J. P., Saliba M., Matsui T., Im S. H., Zakeeruddin S. M., Nazeeruddin M. K., Hagfeldt A., Graetzel M. (2016). Nat. Commun..

[cit20] Abate A., Correa-Baena J.-P., Saliba M., Su’ait M. S., Bella F. (2018). Chem.–Eur. J..

[cit21] Torabi N., Behjat A., Zhou Y., Docampo P., Stoddard R. J., Hillhouse H. W., Ameri T. (2019). Mater. Today Energy.

[cit22] Bhandari S., Roy A., Mallick T. K., Sundaram S. (2020). Mater. Lett..

[cit23] Khalid M., Roy A., Bhandari S., Selvaraj P., Sundaram S., Mallick T. K. (2022). J. Alloys Compd..

[cit24] Sheikh M. S., Roy A., Bhandari S., Mallick T. K., Sundaram S., Sinha T. P. (2020). Mater. Lett..

[cit25] Wang R., Mujahid M., Duan Y., Wang Z.-K., Xue J., Yang Y. (2019). Adv. Funct. Mater..

[cit26] Collavini S., Delgado J. L. (2017). Adv. Energy Mater..

[cit27] Habisreutinger S. N., Leijtens T., Eperon G. E., Stranks S. D., Nicholas R. J., Snaith H. J. (2014). Nano Lett..

[cit28] Chu Q.-Q., Ding B., Peng J., Shen H., Li X., Liu Y., Li C.-X., Li C.-J., Yang G.-J., White T. P., Catchpole K. R. (2019). J. Mater. Sci. Technol..

[cit29] Wu X., Xie L., Lin K., Lu J., Wang K., Feng W., Fan B., Yin P., Wei Z. (2019). J. Mater. Chem. A.

[cit30] Hu R., Zhang R., Ma Y., Liu W., Chu L., Mao W., Zhang J., Yang J., Pu Y., Li X. (2018). Appl. Surf. Sci..

[cit31] Wang S., Jiang P., Shen W., Mei A., Xiong S., Jiang X., Rong Y., Tang Y., Hu Y., Han H. (2019). Chem. Commun..

[cit32] Meng F., Gao L., Yan Y., Cao J., Wang N., Wang T., Ma T. (2019). Carbon.

[cit33] Xu X., Liu Z., Zuo Z., Zhang M., Zhao Z., Shen Y., Zhou H., Chen Q., Yang Y., Wang M. (2015). Nano Lett..

[cit34] Meng F., Liu A., Gao L., Cao J., Yan Y., Wang N., Fan M., Wei G., Ma T. (2019). J. Mater. Chem. A.

[cit35] Rong Y., Liu L., Mei A., Li X., Han H. (2015). Adv. Energy Mater..

[cit36] Batmunkh M., Shearer C. J., Biggs M. J., Shapter J. G. (2015). J. Mater. Chem. A.

[cit37] Cai X., Tang J., Zhao M., Liu L., Yu Z., Du J., Bai L., Lu F., Jiu T., Li Y. (2022). Nano Res..

[cit38] Yu W., Sun X., Xiao M., Hou T., Liu X., Zheng B., Yu H., Zhang M., Huang Y., Hao X. (2022). Nano Res..

[cit39] Sun M., Shu J., Zhao C., Wu J., Guo H., Guo Y., Yin X., Lin Y., Tan Z., He M., Wang L. (2022). ACS Appl. Mater. Interfaces.

[cit40] Yin X., Guo Y., Xue Z., Xu P., He M., Liu B. (2015). Nano Res..

[cit41] Roy A., Ghosh A., Bhandari S., Sundaram S., Mallick T. K. (2020). Build.

[cit42] Bhandari S., Ghosh A., Roy A., Mallick T. K., Sundaram S. (2022). Chem. Eng. J. Adv..

[cit43] Shi B., Duan L., Zhao Y., Luo J., Zhang X. (2020). Adv. Mater..

[cit44] Kwon H.-C., Moon J. (2018). Curr. Opin. Electrochem..

[cit45] Saifullah M., Gwak J., Yun J. H. (2016). J. Mater. Chem. A.

[cit46] Li F. R., Xu Y., Chen W., Xie S. H., Li J. Y. (2017). J. Mater. Chem. A.

[cit47] Bhandari S., Roy A., Ali M. S., Mallick T. K., Sundaram S. (2021). Sci. Rep..

[cit48] Bhandari S., Roy A., Mallick T. K., Sundaram S. (2022). Chem. Eng. J..

