# Supplementary material for: Effect of novel graphitic carbon/NiO hole transporting electrode on the photovoltaic and optical performance of semi-transparent perovskite solar cells
Source: RSC Adv. 2023 Mar 6;13(11):7380–4. doi: 10.1039/d2ra08198a (PMC9987580; doi:10.1039/d2ra08198a)
Supplement: RA-013-D2RA08198A-s001 [file RA-013-D2RA08198A-s001.pdf]

Electronic Supplementary Material (ESI) for RSC Advances.
This journal is © The Royal Society of Chemistry 2023

Effect of Novel Graphitic Carbon/NiO Hole Transporting Electrode on The Photovoltaic and Optical Performance of Semi-transparent Perovskite Solar Cell

Shubhranshu Bhandari,[*a] Sreeram Valsalakumar,[a] Yusuf Chanchangi,[a] Prabhakaran Selvaraj[a,b] and Tapas Mallick[*a]

[a]Environment and Sustainability Institute, University of Exeter, Cornwall, UK, TR10 9FE

[b]Wolfson School of Mechanical, Electrical and Manufacturing Engineering, Loughborough University, UK, LE11 3TU

***Corresponding Author**

Email: s.bhandari@exeter.ac.uk , shubhranshu0094@gmail.com

Theory and calculations

Calculation of transmission of perovskite glazing

The value of luminous transmission or reflection can be acquired from equations 1 and 2.[1]

$$L_v(\alpha) = \frac{\sum_{\lambda=380nm}^{780nm} D_{65}(\lambda)K(\lambda,\alpha)V(\lambda)\Delta\lambda}{\sum_{\lambda=380nm}^{780nm} D_{65}(\lambda)V(\lambda)\Delta\lambda}$$

Luminous transmission or reflection

(1)

For transmission $L_v = \tau_v$ and K (λ, α,) = T (λ, α,); for reflection $L_v = \rho_v$ and K (λ, α,) =R (λ, α,)

$$\tau_s(\alpha) = \frac{\sum_{\lambda=300nm}^{2500nm} S(\lambda)T(\lambda,\alpha)\Delta\lambda}{\sum_{\lambda=300nm}^{2500nm} S(\lambda)\Delta\lambda}$$

Solar transmission (2)

Solar heat gain due to angular transmission

Solar factor (SF), also known as solar heat gain coefficient (SHGC), is an important parameter for glazing as it indicates the amount of transmitted solar energy (SE) and absorbed SE by the

window and reemitted inwards.[2,3] This is the sum of the solar transmittance (τ_s) and entering infrared radiation (q_i) to a building interior.[4]

$$g = \tau_s + q_i \tag{3}$$

$$q_i = \alpha \frac{h_i}{h_i + h_e} \tag{4}$$

$$\alpha = 1 - \tau_s - \rho_s \tag{5}$$

Daylight glare analysis

Building occupant's comfort depends on glare daylight control parameters. Thus, theoretically, glare control potential using perovskite-based BIPV glazing was identified from measured outdoor illuminance on a vertical plane. The glare subjective rating (SR), as shown in equation 6, was evaluated, which certifies the discomfort glare parameter experienced by subject matters when working at a visual daylight task (VDT) positioned against glazing of high or non-uniform luminance.[5]

$$SR = 0.1909 E_v^{0.31} \tag{6}$$

The reason for selecting this index is the engagement of only one photosensor, which can save time and cost. PSC glazing was examined vertically placed, south-facing, having a dimension of 24×24×0.5 (l×w×h) cm in the scale model. This large area mimics perovskite as a large façade while white colour was used for the internal surface with a reflectance of 0.8.[6] Internal vertical illuminance (E_v) was measured at the centre of the room facing the window (worst case).

Colour properties

Quantity and quality of entering daylight through glazing are characterized by correlated colour rendering index (CRI) and correlated colour temperature (CCT). Ideally, transmitted daylight

should maintain CCT from 3000 K to 7500 K while CRI nearing 100 is needed. CRI < 80 is unsuitable for glazing purposes.

CCT was calculated from McCamy's equation 7 [7]

$$CCT = 449n^3 + 3525n^2 + 6823.3n + 5520.33 \quad (7)$$

where

$$n = \frac{(x - 0.3320)}{(0.1858 - y)}$$

$$x = \frac{X}{X + Y + Z}, y = \frac{Y}{X + Y + Z}$$

$$X = \sum_{380nm}^{780nm} D_{65}(\lambda)\ \tau(\lambda)\overline{x}(\lambda)\ \Delta\lambda \quad (8)$$

$$Y = \sum_{380nm}^{780nm} D_{65}(\lambda)\ \tau(\lambda)\overline{y}(\lambda)\ \Delta\lambda \quad (9)$$

$$Z = \sum_{380nm}^{780nm} D_{65}(\lambda)\ \tau(\lambda)\overline{z}(\lambda)\ \Delta\lambda \quad (10)$$

X, Y and Z are termed as tristimulus values which reflect the three-colour perception values of the human eye response, τ_v represents luminous transmittance values, $D_{65}(\lambda)$ is the spectral power distribution of CIE standard illuminant D_{65}, $V(\lambda)$ is the human eye photopic luminous efficiency function and $\Delta\lambda = 10$ nm.

CRI is given by

$$CRI = \frac{1}{8}\sum_{i=1}^{8}\left[100 - 4.6\left\{\sqrt{(U^*_{t,i} - U^*_{r,i})^2 + (V^*_{t,i} - V^*_{r,i})^2 + (W^*_{t,i} - W^*_{r,i})^2}\right\}\right] \quad (11)$$

Tuning into the CIE 1964 uniform colour space system for each test colour is executed using colour space system $W^*_{t,i}$, $U^*_{t,i}$, $V^*_{t,i}$ whereas $W^*_{r,i}$, $U^*_{r,i}$, $V^*_{r,i}$ represents for each test colours, lighted by the standard illuminant D_{65} without the glazing.

$$W^*_{t,i} = 25\left(\frac{100Y_{t,i}}{Y_t}\right)^{1/3} - 17 \tag{12}$$

$$U^*_{t,i} = 13W^*_{t,i}\left(u^{'}_{t,i} - 0.1978\right) \tag{13}$$

$$V^*_{t,i} = 13W^*_{t,i}\left(V^{'}_{t,i} - 0.3122\right) \tag{14}$$

Table S1: Conductivity data of the counter electrode

Printing step	Sheet resistance (Ohm)	Conductivity x10^5 ($S.m^{-1}$)
One-step	25.5	1.5
Two-step	16.5	2.4
Three-step	11.7	4.8

The etching process of FTO glass:

At first, we covered the required area with Zn powder and covered it with 2M HCl. Then waited for the reaction to finish when there was no bubbling. Next, we used a cotton bud, firmly wiped away the etched area of the substrate and rinsed it with DI water.

The cleaning process of etched FTO glass:

Etched FTO Glass was sonicated for 5 minutes in hot (70°C) DI water with the addition of 1% Hellmanex twice. Next, the etched FTO Glass was sonicated for 15 minutes in Isopropyl alcohol, followed by drying. Finally, FTO glass was placed into the UV Ozone cleaner for 15 minutes.

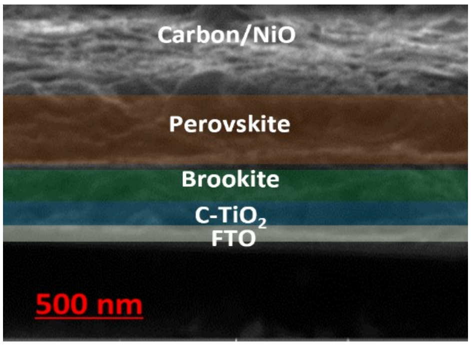

Fig. S1 Cross-sectional SEM of CPSC for the understanding of distinctive layer thicknesses

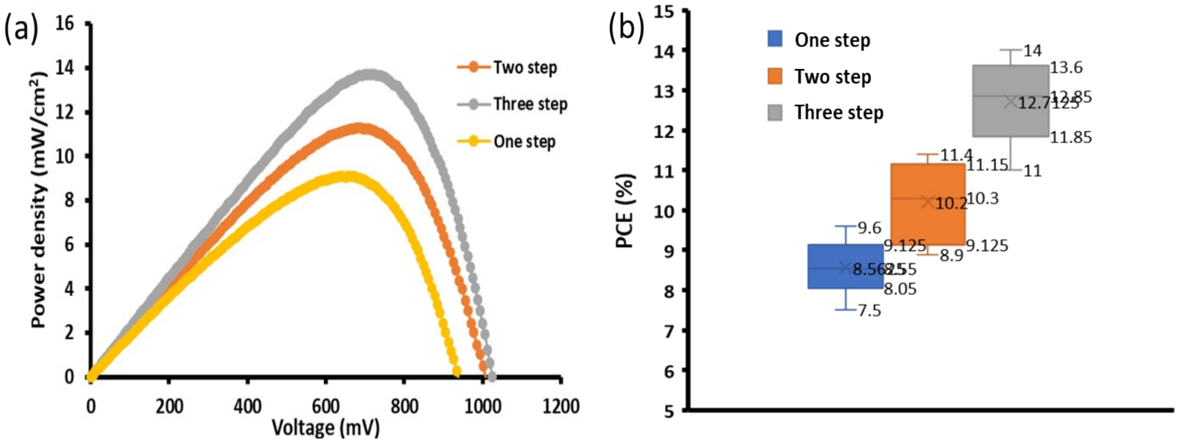

Fig. S2 (a) Power density vs voltage plot of the champion devices from each type and (b) Repeatability test of 6 devices from each set to understand the performances of the devices.

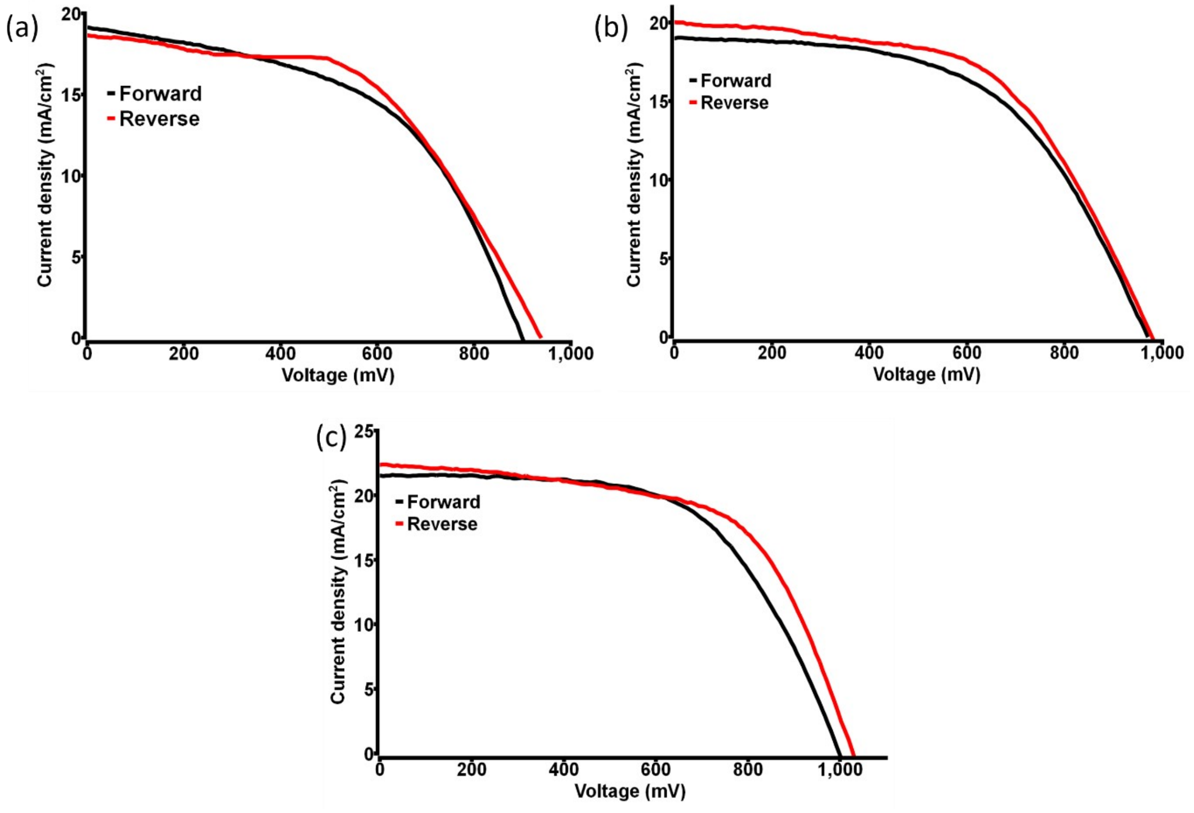

Fig. S3 Hysteresis effect of the (a) one-step, b) two-step, and (c) three-step printing method in forward and reversed biased J-V measurement.

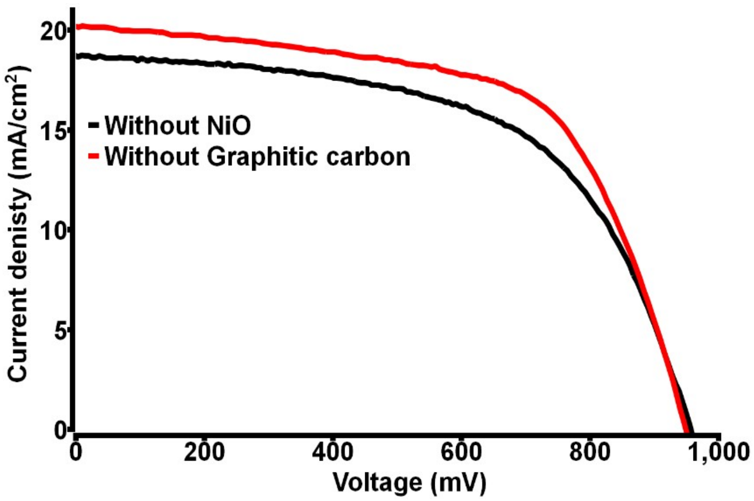

Fig. S4 Photovoltaic performance of devices without NiO and graphitic carbon for three-step coating method.

**Table S2: Average photovoltaic performance and standard deviations of different types of devices under 1sun AM 1.5 for forward and reverse bias with 0.12 cm² active area by masking.**					
Device type		J_{SC} (mA/cm^2)	V_{OC} (mV)	FF (%)	PCE (%)
One-step coating	Forward	18.6±0.4	890±20	49.5±1.5	8.2±1.0
	Reverse	18.4±0.3	910±30	53±2.5	8.5±1.1
Two-step coating	Forward	18.8±0.5	935±35	53±1.8	10±1.2
	Reverse	19.3±0.7	940±45	56.5±2	10.3±1.1
Three-step coating	Forward	21±0.6	950±55	57.5±2.5	12.6±1.1
	Reverse	21.6±0.8	980±50	59±2.2	12.7±1.3

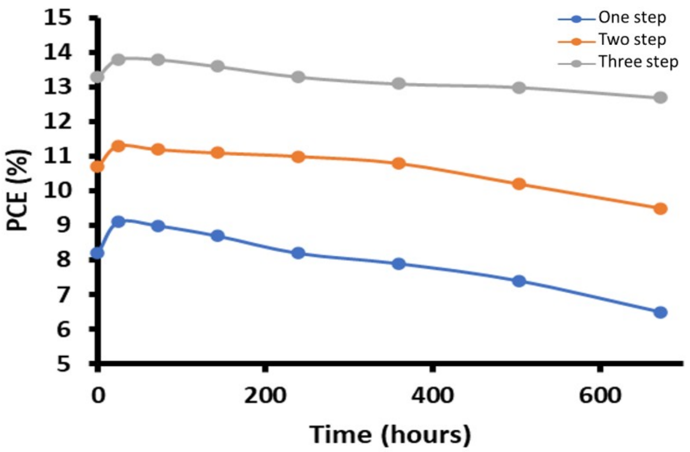

Fig. S5 Stability study of champion devices from each type under ambient conditions kept in the dark

References:

1 A. Ghosh and T. K. Mallick, *Sol. Energy Mater. Sol. Cells*, 2017, 0–1.

2 T. E. Kuhn, *Energy Build.*, 2014, **84**, 388–402.

3 M. C. Singh and S. N. Garg, *Energy Build.*, 2010, **42**, 375–379.

4 A. Ghosh and T. K. Mallick, *Sol. Energy Mater. Sol. Cells*, 2018, **176**, 391–396.

5 E. S. Lee and D. L. DiBartolomeo, *Sol. Energy Mater. Sol. Cells*, 2002, **71**, 465–491.

6 A. Ghosh, B. Norton and T. K. Mallick, *Sol. Energy Mater. Sol. Cells*, 2018, **174**, 572–576.

7 C. S. McCamy, *Color Res. Appl.*, 1992, **17**, 142–144.
